# Analyzing the Role of Gut Microbiota on the Onset of Autoimmune Diseases Using TNF^ΔARE^ Murine Model

**DOI:** 10.3390/microorganisms10010073

**Published:** 2021-12-30

**Authors:** Vivienne Edwards, Dylan L. Smith, Francoise Meylan, Linda Tiffany, Sarah Poncet, Wells W. Wu, Je-Nie Phue, Luis Santana-Quintero, Kathleen A. Clouse, Odile Gabay

**Affiliations:** 1 Division of Biotechnology Review and Research I, Center for Drug Evaluation and Research, U.S. Food and Drug Administration, Office of Biotechnology Products, Office of Pharmaceutical Quality, Silver Spring, MD 20993, USA; ve59@drexel.edu (V.E.); dsmit267@alumni.jh.edu (D.L.S.); Linda.Tiffany@fda.hhs.gov (L.T.); sarah.poncet@fda.hhs.gov (S.P.); kathleen.clouse@fda.hhs.gov (K.A.C.); 2 Translational Immunology Section, NIH, National Institute of Arthritis and Musculoskeletal and Skin Diseases, Bethesda, MD 20892, USA; meylanf@mail.nih.gov; 3 Facility for Biotechnology Resources, Center for Biologicals Evaluation and Research, U.S. Food and Drug Administration, Silver Spring, MD 20993, USA; wells.wu@fda.hhs.gov (W.W.W.); jenie.phue@fda.hhs.gov (J.-N.P.); 4 U.S. Food and Drug Administration, Center for Biologics Evaluation & Research, Office of Biostatistics and Epidemiology, HIVE, Silver Spring, MD 20993, USA; Luis.santana-quintero@fda.hhs.gov; 5 U.S. Food and Drug Administration, Center for Drug Evaluation and Research, Office of New Drugs, Office of Hematology and Oncology Products, Silver Spring, MD 20993, USA

**Keywords:** gut microbiome, dysbiosis, Fecal Matter Transplants/Fecal Matter Transplanted (FMT), auto-immune diseases

## Abstract

Very little is known about disease transmission via the gut microbiome. We hypothesized that certain inflammatory features could be transmitted via the gut microbiome and tested this hypothesis using an animal model of inflammatory diseases. Twelve-week-old healthy C57 Bl/6 and Germ-Free (GF) female and male mice were fecal matter transplanted (FMT) under anaerobic conditions with TNF^ΔARE−/+^ donors exhibiting spontaneous Rheumatoid Arthritis (RA) and Inflammatory Bowel Disease (IBD) or with conventional healthy mice control donors. The gut microbiome analysis was performed using 16S rRNA sequencing amplification and bioinformatics analysis with the HIVE bioinformatics platform. Histology, immunohistochemistry, ELISA Multiplex analysis, and flow cytometry were conducted to confirm the inflammatory transmission status. We observed RA and IBD features transmitted in the GF mice cohort, with gut tissue disruption, cartilage alteration, elevated inflammatory mediators in the tissues, activation of CD4/CD8+ T cells, and colonization and transmission of the gut microbiome similar to the donors’ profile. We did not observe a change or transmission when conventional healthy mice were FMT with TNF^ΔARE−/+^ donors, suggesting that a healthy microbiome might withstand an unhealthy transplant. These findings show the potential involvement of the gut microbiome in inflammatory diseases. We identified a cluster of bacteria playing a role in this mechanism.

## 1. Introduction

As research on the immune system has expanded over the last decade, the gut microbiome has been increasingly identified as a key player. The mechanisms involved in its homeostasis or imbalance and its impact on diseases, treatments, and interactions with the mucosal immune system are described today more and more [1,2,3]. A bacterial or viral origin of chronic inflammatory and auto-immune diseases has been suspected for decades, but it is still unclear whether one specific bacteria or virus could trigger disease etiology. However, a consensus of bacteria and viruses interacting together is becoming a more plausible idea. Although we are still in the observational portion of this discovery, more research is emerging that reveals the specific mechanisms underlying the link between bacteria, their metabolites, and the immune system [2,3] The gut microbiome, comprised of 95% bacteria, fungi, viruses and other microorganisms, is in physiological homeostasis in healthy humans [4,5]. An imbalance, called dysbiosis, results in pathophysiological mechanisms and events leading to metabolic changes and the initiation of disease states [6]. Interesting correlations, such as the brain-gut axis and joint-gut axis, have been uncovered, shedding light on our understanding of how the human body’s systems function and interact as a whole [7,8]. The expansion and exponential increase in auto-immune diseases over the last few decades are inversely correlated to the decrease of infectious diseases, due to increased hygiene, better health care and vaccinations [9]. Among autoimmune and inflammatory diseases, rheumatologic diseases such as rheumatoid arthritis (RA), lupus, spondyloarthritis and psoriatic arthritis are prevalent. Inflammatory Bowel Diseases (IBD), such as Crohn’s disease and ulcerative colitis, are also predominant and growing in the population. In this context, some discoveries have been made in the joint-gut axis, leading to new therapeutic targets and potential novel drug development in the future. Jose Scher et al. intensively worked on RA and the gut microbiome and have shown involvement of the bacteria *Prevotella copri* in the etiology of RA [10]. The potential for treatment by fecal transplant has also been investigated and preliminary data in the brain-gut axis have shown some promising results in Alzheimer and Parkinson diseases [11,12,13], as well as in IBD, by autologous fecal microbiota transplantation [14]. Although FMT has been utilized in research as a potential therapeutic, it has not been looked at in many studies as a tool of disease transmission; FMT has been investigated in obesity and Parkinson’s disease [15,16]. One group has shown that transplanting the fecal microbiota of each member of a discordant twin pair—one obese and the second lean—into separate groups of GF mice permits the donors’ communities to be replicated, variant features to be identified, and transmission of the phenotype to be performed. The differences were correlated with changes in the fermentation of short chain fatty acids, metabolism of amino-acids, and microbial transformation of bile acid species [17]. We hypothesized that transplanting fecal matter from sick mice into germ free (GF), as well as conventional, healthy (C) mice as their control, could partially transmit some inflammatory disease features. We propose that an already present, healthy microbiome in C mice could conceivably prevent this phenomenon.

As an animal model, we chose the TNF^ΔARE+/^^−^ mouse model, largely used to study inflammatory diseases [18,19,20,21,22]. TNF^ΔARE^ transgenic mice overexpress TNF and develop inflammation in tissues and organs, and spontaneously exhibit rheumatoid arthritis (RA) and inflammatory bowel disease (IBD) [23]. TNF^ΔARE−/−^ mice develop severe systemic inflammation and physical disability as they age and do not survive beyond 1 to 2 months of age. However, the heterozygous mice TNF^ΔARE/Het^, or TNF^ΔARE+/^^−^, develop a similar phenotype and are also disabled as they age but live a longer life, which is why we chose to use this model.

## 2. Materials and Methods

Animals: 12-week-old GF and conventional SPF C57 Bl-6 mouse groups (*n* = 16 females and *n* = 20 males) were provided by Taconic (Taconic Bioscience, Inc., Rensselaer, NY, USA). TNF^ΔARE^ mice were provided by Prof. Fabio Cominelli, Case Western Reserve University, Cleveland, OH, and housed in the NIH Bdg 10 animal facility. TNF mRNA contains a repeated oligonucleotide AU-rich motif in its 3′ untranslated region, implicated in post-transcriptional and translational regulation of TNF synthesis. Mice bearing an endogenous deletion of the 3′-AU-rich region in the gene encoding TNF possess high circulating levels of TNF protein [24,25]. Fecal Matter Transplantation, FMT was performed within the FDA animal facility. One mouse of each gender underwent FMT with one mouse donor from the same gender. GF mice were housed in a sterile semirigid isolator (SRI) in a dedicated GF room (Park Bioservices, LLC, Groveland, MA, USA) as described in Gabay et al. [1,26]. After FMT, GF mice were kept in a gnotobiotic environment in a new isolator for 12 weeks, and co-housed to minimize unwanted variations in the gut microbiome. Fecal samples were collected every other week for monitoring the colonization and the stabilization of the new microbiome. Conventional mice were housed under standard conditions. All mice were co-housed and fed the same diet of autoclaved food and water, housed in autoclaved bedding and kept under standard temperature and light. All animal procedures and the Animal Study Proposal (ASP) were reviewed and approved by the U.S. Food and Drug Administration (FDA) Institutional Animal Care and Use Committee (IACUC).

FMT Preparation and Processing: Three fecal pellets from TNF^ΔARE+/^^−^ mice or conventional Bl/6 healthy mice were snap frozen in 150 µL PBS and stored at −80 °C. To preserve as much anaerobic bacteria as possible, all preparations were conducted inside an anaerobic chamber (Type B Coy vinyl anaerobic chamber, Coylab.com, Grass Lake, MI, USA) under sterile conditions. An additional 150 µL of anaerobic sterile PBS was added to each tube in the anaerobic chamber, before samples were homogenized with a sterilized Biomasher II (DiagnoCine, Hackensack, NJ, USA) and centrifuged. Then, 150 µL of supernatant was aspirated into a sterile single wrapped 1 mL Monoject Tuberculin Syringe (Covidien, Jersey City, NJ, USA) with a sterile animal feeding needle (Cadence Science, Cranston, RI, USA). Syringes were immediately used for oral gavage. For use in the GF isolator, standard SOP and procedures were used as per protocol described in Gabay et al. [26].

Mice Dissection: Mice were euthanized using CO_2_ and processed as described in Gabay et al. [1] Briefly, using surgical scissors, a midline cut was made in the abdomen exposing the gut. The small intestine was removed, and four 1 cm pieces of the gut were cut at the same median between the stomach and the cecum. One piece was placed in 4% paraformaldehyde, while the remaining three were snap frozen for protein analysis. The remaining gut was processed to isolate lamina propria lymphocytes for further flow cytometry and Lamina Propria immune cell analysis. A colon fecal sample was removed and snap frozen for DNA analysis. A knee and hip were dissected from each mouse and placed in 4% paraformaldehyde for histological staining and immunohistochemistry (American Histolabs, Gaithersburg, MD, USA; HistoServ, Gaitherburg, MD, USA). Spleens were collected and processed to isolate cells for analysis by flow cytometry.

Genomic DNA Extraction from Fecal Samples: Genomic DNA was extracted using the Power Fecal DNA isolation kit (Mo-Bio, Carlsbad, CA, USA) following the manufacturer’s instructions. Each fecal pellet was cut to weigh 0.025 g. Volumes of the solution used to dissolve the pellets were adjusted to this weight. Final elution was done in 80 μL of the kit elution buffer.

16S rRNA Library Preparation and Sequencing: 16S rRNA library preparation and sequencing were conducted following the Illumina’s 16S Metagenomic Sequencing Library Preparation Guide Rev. B (Illumina Inc., San Diego, CA, USA), reported in our previous study [1]. Briefly, microbial genomic DNA samples were normalized to an equal concentration and 2.5 μL of microbial genomic DNA samples were used to create a single amplicon using custom primer pairs specifically targeting variable V3 and V4 regions of the 16S rRNA gene. During the amplification, Illumina sequencing adapters and dual-index barcodes were added to the amplicons. The PCR products were cleaned up and dual indices were attached using Illumina Nextera XT Index kit (Illumina Inc., San Diego, CA, USA) and Kapa HiFi HotStart ready Mix. The final libraries were cleaned up again, quantified and normalized using Bioanalyzer DNA 1000 chip (Agilent Technologies Inc., Santa Clara, CA, USA) and Qubit DNA assay (Life Technologies Corp., Eugene, OR, USA). Libraries were pooled, denatured, and loaded on the Illumina MiSeq and sequenced paired end (2 × 300 cycles) using a MiSeq Reagent Kit v3 (600 cycles). The MiSeq sequencing FASTQ files were generated using MiSeq Reporter software

Bioinformatics analysis of sequencing data using the High-Performance Integrated Virtual Environment (HIVE) platform [27]. The CBER HIVE platform was used to transfer data and perform the bioinformatics analysis, quality control on the sequencing data, as previously described in Gabay et al. [1]. The FASTQ files were uploaded into the cloud-based system. Upon data upload, HIVE automatically calculates basic quality control (QC) metrics of sequencing runs, using them to determine if an individual sequence run worked well, as previously described in Gabay et al. [1]. Optimized metagenomic pipeline is then executed using the CensuScope pipeline [28]. The Silva Bacteria database (release 102) with 14,956 sequences [29] is used as a reference to align the short reads and identify reads to specific genomes using the NCBI taxonomy database [30] to find the taxonomic identifier. CensuScope has been parallelized in HIVE. The HIVE CensuScope tool determines the taxonomic composition of a metagenomic sample and provides users with standard formatted reports of hits classified into species or higher taxonomic nodes. Mapping the sequence data to the Silva Bacteria database is done using the basic local alignment search tool (BLAST) [31] with the following parameters: (-task megablast -evalue 1 × 10^−6^ -best_hit_score_edge 0.1 -best_hit_overhang 0.1 -num_alignments 1 -num_descriptions 1). Our analysis using CensuScope with the suggested parameters (1000 randomly picked reads and 50 iterations) yield a statistical power of >99% for detecting taxa, with an estimated error of 3.4% according to the publication cited.

Regularized Fisher’s linear discriminant analysis (RLDA) was performed to detect bacterial populations to identify main discrimination markers among the groups. In Figures 5 and 6, a 3-D plot depicts the results from RLDA, delineating the murine gut microbial profile through vectorization in three-dimensional space. Each marker is representative of one murine sample, with different groups being assigned unique colors. To obtain the 3-D plot, a JavaScript library (Three.js) was implemented to generate interactive graphs that can be manipulated to preview data from various angles, change colors and zoom in/out for a detailed view, also allowing us to save the 3-D plot in PNG format. Our raw data are to be transferred to the NCBI microbiome database registry depository, in the Sequences Read Archives (SRA): https://www.ncbi.nlm.nih.gov/sra/ (accessed on 15 December 2021).

Luminex Analysis: Analysis was conducted using mouse serum and gut tissue samples. Gut proteins were extracted using Tissue Protein Extraction Reagent (ThermoFisher, Rockford, IL, USA) with HALT Protease Inhibitor Cocktail (ThermoFisher, Rockford, IL, USA) in PowerBead Tubes, Metal 2.38 mm (Qiagen, Hilden, Germany). Tissue was disturbed in the incubating/cooling shaker (VWR, Radnor, PA, USA) at 6000 rpm and supernatant stored in new Eppendorf tubes. Serum and gut protein samples were then prepared using the Bio-Plex Pro Reagent Kit V (Bio-Rad, Hercules, CA, USA), Pro-Mouse Cytokine TNF-α Set, IL-1β Set, IL-6 Set, and IL-17A Set (BioRad, Hercules, CA, USA) with Pro-Mouse Cytokine Standards Group I (BioRad, Hercules, CA, USA). Multiplex experiments were conducted as per manufacturer instructions on the BioRad BioPlex 200 Luminex (Austin, TX, USA).

Histology and Immunohistochemistry: Knees and hips were dissected and fixed in 4% paraformaldehyde (PFA) for 36 h, then conserved in 70% ethanol. Joints were decalcified in a solution containing formic acid and paraformaldehyde, after which joints were dehydrated and embedded in paraffin. Gut was dissected, fixed, and conserved using the same method. Sections of 5 μm were cut and stained with Safranin’O/light green for the joints and Periodic Acid Shiff (PAS) for the gut. Unstained slides were used to perform Immuno-histochemistry with Rabbit Polyclonal Anti-TNF-alpha Antibody, Rabbit Polyclonal Anti-IL-1 beta/IL-1F2 Antibody, and Mouse Monoclonal Anti-IL-6 Antibody (Novus Biologicals, Littleton, CO, USA).

Flow Cytometry: After euthanasia, spleens were removed and then smashed with a 3 mL Luer–Lok tip syringe (BD, Franklin Lakes, NJ, USA) through a 40 μm Nylon cell strainer (Falcon, Corning, NY, USA) in a 50 mL tube containing RPMI-1640 complete media (BioWhittaker Lonza, Walkersville, MD, USA) enriched with sodium pyruvate (Gibco, Grand Island, NY, USA) and Penicillin Streptomycin (Gibco, Grand Island, NY, USA). Cells were extracted using 1.2 mL ACK Lysis Buffer (Gibco, Grand Island, NY, USA) for one minute for the removal of red blood cells and resuspended in complete medium. Cells were then stained for flow cytometry using FOXP3/Transcription Factor Staining Buffer Set (Invitrogen, eBioscience, Eugene, OR, USA), with LIVE/DEAD™ Fixable Aqua Dead Cell Stain Kit (Invitrogen, Eugene, OR, USA), lymphocytes using anti-CD3e Percy5.5 (BD Biosciences; Franklin Lakes, NJ, USA) and CD4 and CD8 T-cells using anti-CD4 V450 (BD Biosciences; Franklin Lakes, NJ, USA) and anti-CD8 FITC (BD Biosciences; Franklin Lakes, NJ, USA). Stained cells were immediately run on Fortessa X20 (BD Bioscience; Franklin Lakes, NJ, USA) and analyzed using FlowJo V10.

Statistical analysis: Results were analyzed using ANOVA in JMP 16 and unpaired two-sample-t-test analysis GraphPrism software. The green diamonds created by the software JMP16 represent the mean for each group (medium line) as well as the interval estimates for each group (top and bottom points). The groups are represented in the X axis and the percentage of different phyla present in the gut are shown in the Y axis. Data are presented as mean ± SEM. A value of *p* < 0.05 was considered statistically significant.

Study design: we provide a summary of our study design in Supplemental Figure S1.

## 3. Results

### 3.1. Behavioral and Physical Changes

**Figure 1:**
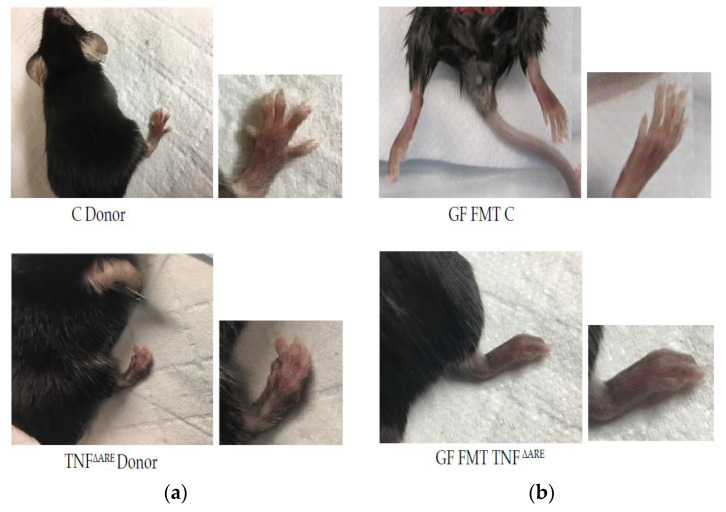
Macroscopic in vivo phenotypes (**a**)—Representative pictures of donor mice. Top: male conventional donor control (C), *n* = 9. Bottom: male TNF^ΔARE+/−^ sick donor with enlargement of the right lower limb (*n* = 11). TNF^ΔARE+/−^ donor exhibits a degenerative arthritis with deformation and swelling phenotype compared to control. (**b**)—Picture of GF mice, that received FMT from C or TNF^ΔARE+/−^ donor. Top: control GF mice FMT with C donor which exhibit a phenotypically normal paw. Bottom: GF mice FMT with TNF^ΔARE+/−^ exhibiting a phenotype with deformation and swelling similar to the TNF^ΔARE+/−^ donor. Females show the same phenotypic pattern and pictures are representative of the entire colony. Histological examination of the gut and the joints of these GF male and female mice subjected to FMT with TNF^ΔARE+/^^−^ fecal matter, shows a degradation of the knee joints (clefts, debris, tears and loss of cartilage) and damages to the gut (thickening of the wall, disorganization of the villi, cell infiltration) compared to the group of male and female GF mice FMT with C mice, panels, see Figure 2a,c. Immunohistochemistry (IHC) was then conducted using anti-TNF antibody. In GF mice FMT with TNF^ΔARE+/^^−^, as compared to GF mice FMT with healthy microbiome, IHC showed significant pro-inflammatory cytokine signature in tissues, particularly in the gut. We can localize the TNF staining in the gut epithelial layer cells and in the chondrocytes of the cartilage surface (Figure 2, panels (b,d) Results were similar between males and females).

After fecal microbiome transplants (FMT) in the germ-free colony, GF mice were kept co-housed in a gnotobiotic environment in a new isolator for 12 weeks. Colonization of the gut was monitored every other week by collecting fecal samples. We started to notice behavioral changes at week 8 in the mice fecal matter transplanted (FMT) with TNF^ΔARE+/−^ mice as compared to their counterpart controls, GF fecal matter transplanted (FMT) with C, with mice becoming increasingly aggressive over time, exhibiting defensive postures and an increase in biting behavior. In addition to the behavioral changes in GF mice FMT with TNF^ΔARE+/^^−^ mice, we found physical changes consistent with IBD and RA: scruffy hair and protective position suggesting pain in the belly, blood in their feces when collecting fecal samples, which became more and more difficult to obtain, as well as deformation in their paw shape (Figure 1, panel (a) and (b)). Comparatively, we did not notice a specific behavioral or physical change in GF mice FMT with C possessing a conventional, healthy microbiome, which were housed in the same isolator and did not show pictures of those phenotypical control mice. Mice were transplanted with only adult donors, and a potential transmission with early FMT was not tested.

**Figure 2:**
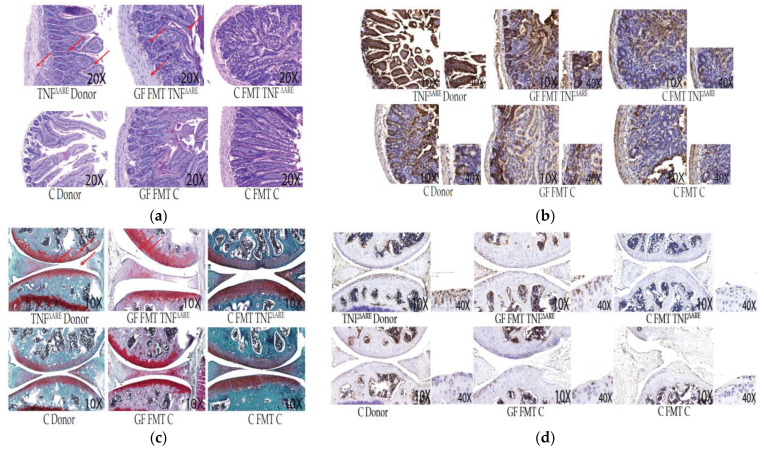
Descriptive histopathology and immunohistochemistry. Our data shown are representative of ~90% of similar results in FMT mice Panel (**a**)—Histological intestinal sections. Representative images were obtained at 10× magnification as indicated and stained with Periodic Acid Shiff (PAS). Arrows represent thickening of the wall and disorganization, blunting of the villi and cell infiltrations. GF mice FMT with C donor and conventional C mice do not display histological differences or pathology. GF mice FMT with TNF^ΔARE+/−^ donors exhibit similar histopathology to the donors themselves. Panel (**b**)—Intestinal immunohistochemistry sections. Representative images were obtained at 10× magnification as indicated. Slides were stained with anti-TNF polyclonal antibody. Massive TNF production is observed in TNF^ΔARE+/−^ donor intestines and GF mice FMT with TNF^ΔARE+/^^−^ donors compared to their control counterparts. Enlargement of the intestinal epithelial cell layer at 40× magnification indicates that TNF production is localized in epithelial cells in TNF^ΔARE+/^^−^ donors and GF FMT with TNF^ΔARE+/^^−^ donors. Other immunohistochemistry cuts display a normal baseline level of TNF, not localized in the epithelial cells. Panel (**c**): Histological knee sections. Representative images were obtained at 10× magnification as indicated and stained with Safranin’O/light green. TNF^ΔARE+/−^ donor sections and GF mice FMT with TNF^ΔARE+/^^−^ donors show features of joint degradation indicative of arthritis: Arrows point out clefts, debris, tears and loss of glycosaminoglycans. These features are not observed in controls, C donors, GF FMT with C donors and C mice FMT with either donors. Panel (**d**): Knee immunohistochemistry sections. Slides were stained with anti-TNF polyclonal antibody. Chondrocyte nuclei are stained in blue. Enlargement of the joint surface at 40× magnification indicates that TNF production is localized in chondrocytes in TNF^ΔARE+/−^ donors knee cartilage and in GF mice FMT with TNF^ΔARE+/^^−^. TNF production is not observed in chondrocytes from control mice.

### 3.2. Inflammation

**Figure 3:**
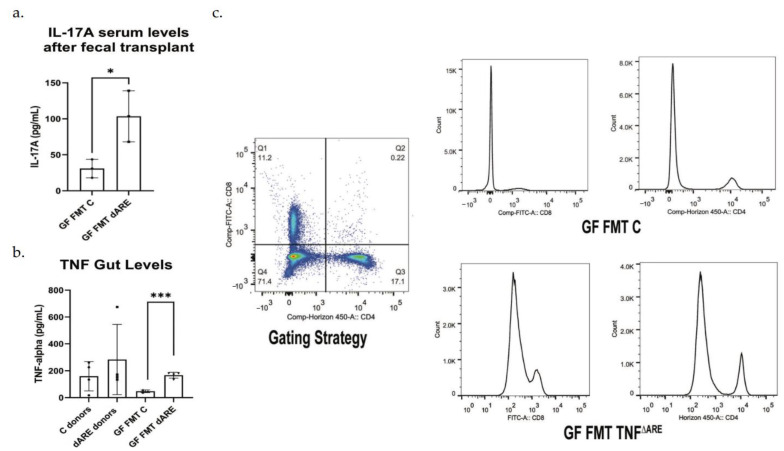
Proinflammatory cytokines measurement and inflammation evaluation by flow cytometry. GraphPad Prism was used to generate the bar plots, that shows the mean of data and error bars show standard deviation. Stars represent significance (* for *p* < 0.05 and *** for *p* < 0.001). Multiplex ELISA Assay (Luminex). (**a**) Multiplex assay was conducted on the serum, with antibodies against TNF, IL-1β, IL-6 and IL-17. Results show a serological statistically significant (*p* = 0.0290) increase in IL-17A level in serum of GF mice FMT with TNF^ΔARE+/^^−^ donors (*n* = 6) compared to their controls, GF mice FMT with C donors (*n* = 3). No significant difference was detected in serum TNF, IL-1 β and IL-6 levels. Results were similar between males and females and were representative of the colony. (**b**) The multiplex assay was conducted at the tissue level after gut protein extraction. TNF levels were significatively elevated in TNF^ΔARE+/^^−^donors (*n* = 4) compared to control donors (*n* = 4). TNF levels were below the limit f detection in healthy control mice donors. The levels of TNF were significatively elevated in GF mice FMT with TNF^ΔARE+/^^−^ (*n* = 4) compared to GF mice FMT with C (*n* = 3, *p* = 0.0004). (**c**)—Flow cytometry was conducted on spleen cells using antibodies directed against CD4 and CD8. After gating on lymphocytes, results show an activation and expansion of CD4+ and CD8+ T cells in GF mice FMT with TNF^ΔARE+/^^−^ when compared to their counterpart controls, suggesting an immune response initiation and inflammatory status initiation. Data shown from *n* = 4 in each group.

We quantified the pro-inflammatory cytokines TNF and IL-17 and found a level of local basal inflammation in the gut tissue of C mice donors and a higher trend in TNF^ΔARE+/^^−^ donors, with a significant elevated level of TNF in the mice FMT with TNF^ΔARE+/^^−^ compared to their counterparts (Figure 3 panel (b)) Additionally, we found a moderate elevation of the systemic inflammatory marker, IL17A, in the serum of these mice (Figure 3, panel (a).

After obtaining immune cells from the spleens, we gated on lymphocytes, then gated on CD4/CD8 positive T-cells and found an activation and expansion of those cells in the GF mice FMT with TNF^ΔARE+/^^−^compared to their counterpart, healthy control mice, from 11.2% and 17.1% to 18.8% and 20.3%, respectively (Figure 3, panel (c)).

The group of control mice FMT with either control or TNF^ΔARE+/^^−^ donors do not exhibit any change in IL-17 levels (under the level of detection) or TNF and there was no peak of T cells activation shown by flow cytometry.

### 3.3. Colonization

**Figure 4:**
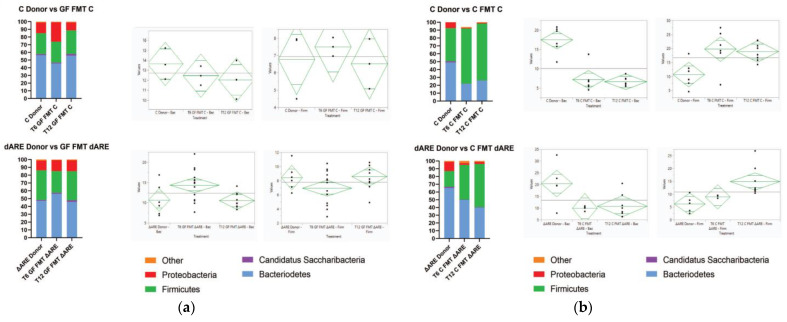
Colonization overtime of the GF and Control colonies after FMT. Colonization at phyla level. Graph Pad Prism was used for generating the stacked bar graphs and the diamond plots were generated by JMP16 software. They represent the confidence interval of each group. The medium line is the mean of the sample group. The Y axes are the CI range for each group. Panel (**a**) Phylogenic results after NGS and HIVE bioinformatics analysis showing the gut microbial profile at the phyla level 6 weeks (T6) and 12 weeks (T12) after GF mice were fecal transplanted from C (top) or TNF^ΔARE+/−^ (bottom). At T6, we observe a reshuffling of the microbiome profile in both GF mice FMT with C donors (*n* = 3) and GF mice FMT with TNF^ΔARE+/−^ donors (*n* = 14). At T12 weeks, the microbiome profile of recipient mice is completely normalized relative to their donors, both in GF mice FMT with control donors (*n* = 3) and with TNF^ΔARE+/−^ donors (*n* = 10). ANOVA statistical analysis shows no significant difference between T12 mice and their respective donors (*p* > 0.05), for both the Bacteroidetes and Firmicutes phyla, for GF mice FMT with either C or TNF^ΔARE+/−^. Panel (**b**) Phylogenic results showing healthy control mice FMT with C (top) or TNF^ΔARE+/−^ (bottom) donors, 6 weeks (T6) and 12 weeks (T12) after fecal transplant. At T6, we observe a reshuffling of the microbiome profile in both C FMT with C donors (*n* = 6) or C FMT with TNF^ΔARE+/−^ donors (*n* = 4). By T12, the microbiome profile of the recipient mice is significatively different from the donor, for both C mice FMT with C donors (*n* = 8) and C mice FMT with TNF^ΔARE+/−^ donors (*n* = 8). ANOVA statistical analysis shows a significant difference at T12 between C Donors and C mice FMT with C donors, for both the Bacteroidetes phyla (*p* < 0.0001) and Firmicutes phyla (*p* < 0.0001). ANOVA statistical analysis shows a significant difference at T12 between TNF^ΔARE+/−^ donors and C mice FMT with TNF^ΔARE+/−^ donors, for both the Bacteroidetes phyla (*p* = 0.0121) and Firmicutes phyla (*p* = 0.0439).

After investigating the gut microbiome taxonomic profile of the TNF^ΔARE+/^^−^ and healthy conventional donors, we examined the colonization in two different colonies: GF mice transplanted with TNF^ΔARE+/^^−^ donors, compared to GF mice transplanted with healthy controls. After 6 weeks post-FMT, the gut microbial profile of GF mice FMT with either donor stabilized, normalizing to a profile similar to their respective donors’ (Figure 4).

In C mice FMT with either control donor or TNF^ΔARE+/^^−^ donor, we observed no phenotypical behavioral or physical changes in the mice. Histological examination did not reveal any difference in the joints or the gut of C mice receiving FMT from either donor, when compared to the healthy C donors (Figure 2, panel (a) and (c)). Immunohistochemistry shows a basic low level of gut inflammation in all mice. There was no increase in inflammatory markers analyzed by multiplex in the gut tissue, with nondetectable levels for all inflammatory markers, and no systemic inflammation observed in both C mice FMT with C donors, or TNF^ΔARE+/^^−^ donors (Figure 2, panel (b) and (d)). The taxonomic analysis shows a similar gut profile of C mice before and after receiving FMT from healthy or sick donors; there is no observed transmission of the TNF^ΔARE+/^^−^ donor microbiome profile in C mice recipients. Results were similar in males and females. When C conventional Bl/6 mice exhibiting a healthy microbiome receive FMT with TNF^ΔARE+/^^−^ donors, 12 weeks post-FMT, their overall gut microbial profile remains identical to their pre-FMT profile, regardless if the FMT is done with TNF^ΔARE+/^^−^ donor microbiota or C healthy control microbiota (Figure 4, panel (a) and (b)).

### 3.4. Taxonomy: Phyla Level

**Figure 5:**
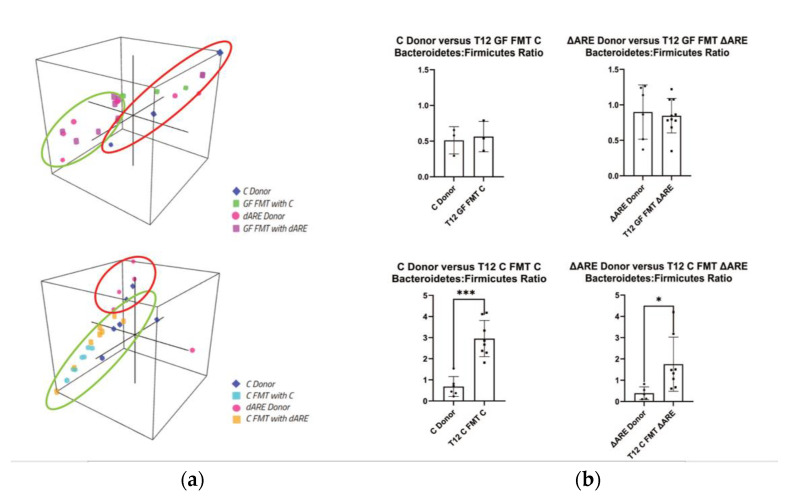
3D visualization of phyla communities in FMT colonies and quantification of Bacteroidetes/Firmicutes ratio. GraphPad Prism was used to generate the bar plots that shows the mean of data and error bars show standard deviation. Stars represent significance (* for *p* < 0.05, ** for *p* < 0.01 and *** for *p* < 0.001). Taxonomy panel at Phyla level. Panel (**a**) 3D representation of clustering at the Phyla level. Top: Donors (*n* = 9) and GF mice FMT with Controls or TNF^ΔARE+/−^ (*n* = 13). Clustering indicates that GF recipient mice are localized closely to their respective donor mice together. Bottom: Donors (*n* = 11) and GF mice FMT with Controls or TNF^ΔARE+/−^ (*n* = 16)—Clustering indicates that C donors, C mice FMT with C, and C mice FMT with TNF^ΔARE+/−^ are localized apart from TNF^ΔARE+/−^ donors. TNF^ΔARE+/−^ donors are clustered together, but far apart from other groups, indicating a unique 3D localization and microbial profile. Panel (**b**) Firmicutes/Bacteroides ratio. Top: Comparison between donors and GF mice FMT with their respective donors. No statistical difference is observed in the B/F ratio for C donor (*n* = 3) vs. GF FMT C (*n* = 3) and for TNF^ΔARE+/−^ donor (*n* = 6) and GF FMT TNF^ΔARE+/−^ (*n* = 10). Bottom: Comparison between donors and Control conventional, healthy mice recipients. Two-way student’s *t*-test statistical analysis indicates a significant difference (*p* = 0.0258) in C mice FMT with C (*n* = 8) compared to C donors (*n* = 6), and a significant difference (*p* = 0.040) in C mice FMT with TNF^ΔARE+/−^ (*n* = 8) compared to TNF^ΔARE+/−^ donors (*n* = 5).

**Figure 6:**
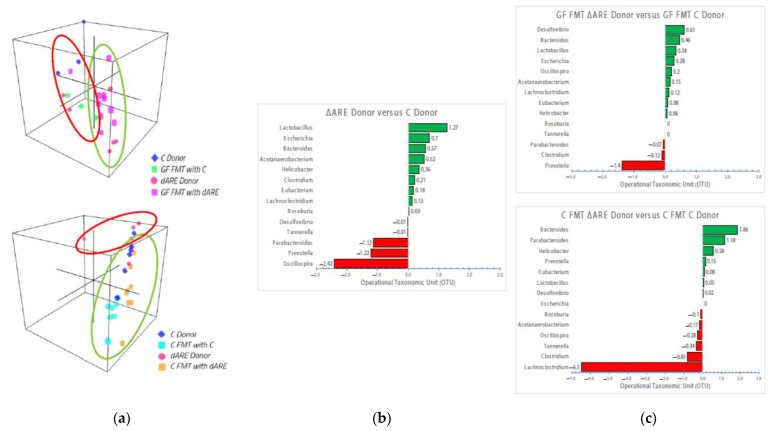
3D visualization of genus communities in FMT colonies and bidirectional bar chart of the 14 genera identified in the disease transmission. Taxonomy panel at the Genus level. Panel (**a**) 3D representation of clustering at the genus level. Top: GF mice (*n* = 13) 3D localization is clustered closely to their respective donors (*n* = 9), similar to what is observed at the phyla level. Bottom: TNF^ΔARE+/−^ donors (*n* = 5) cluster separately from C donors (*n* = 6) and C mice FMT with either donor (*n* = 11), similar to the observations at the phyla level. Panel (**b**) Bidirectional bar chart. BLAST TaxID (OTU) representation of the genera proportion in the donor mice (*n* = 9). Panel (**c**) Top: BLAST TaxID (OTU) representation of the genera proportion in GF mice (*n* = 13). Bottom: BLAST TaxID (OTU) representation of the genera proportion in Control mice (*n* = 16). These 14 genera were identified as the major key players, exhibiting significant changes in quantity between groups.

A three-dimensional (3D) representation of the taxonomic data, at both a phyla and genus level, confirms transmission of the TNF^ΔARE+/−^ donor microbial profile to GF mice but not to C mice. Clustering is seen between GF mice and their respective donors, but C mice FMT with either C or TNF^ΔARE+/^^−^ donors have distinct localization in 3D space (Figure 5, panel (a), Figure 6, panel (a)). A healthy microbiome is characterized by a stable ratio between the two major phyla present in the gut, Bacteroidetes and Firmicutes [32]. A ratio of Bacteroidetes to Firmicutes conveys a big-picture view of the gut health. Although GF mice exhibited ratios similar to their respective donors, C mice FMT exhibited statistically significant different ratios compared to their respective donors (Figure 5, panel (b)).

### 3.5. Taxonomy: Genus Level

After observation of the dysbiosis in TNF^ΔARE+/^^−^donors and GF FMT mice transplanted with sick donors, we looked deeper into the emergent Phyla, performing BLAST TaxID analysis and reanalyzing the data at the genus level, where we identified 14 key players exhibiting increased prevalence in sick donors and GF mice FMT with sick TNF^ΔARE+/^^−^ donors, compared to their respective controls (Figure 6, panel (b) and (c)). Interestingly, we found the same emergent key players already identified in our previous publication linked with TNF mechanisms and functions [1], that have also been identified by others in an inflammatory context [10]: *Lactobacillus*, *Escherichia*, *Bacteroides* and *Parabacteroides, Actetanaerobacterium*, *Helicobacter*, *Clostridium* and *Lachnoclostridium*, *Eubacterium*, *Roseburia*, *Prevotella* and *Oscillospira* are the microorganisms with the major amplitude changes observed in BLAST TaxID (Figure 6, panel (c)). *Prevotella*, *Lactobacillus*, *Clostridium* and *Lachnoclostridium, Bacteroides and Parabacteroides* were consistently identified as having major amplitude differences between groups, indicating that they may participate in crosstalk and may be involved in a complex mechanism, working together to induce an inflammatory environment through yet to be identified mediators.

## 4. Discussion

Our main observation in this study is the transmission of an inflammatory phenotype in germ-free mice transplanted with fecal matter from genetically engineered murine donors exhibiting RA and IBD, particularly in the gut. The dysbiosis observed in murine donors showing inflammation was transmitted to germ-free mice, but not to conventional healthy mice that had a healthy microbiome, whether they were FMT with healthy C or sick murine TNF^ΔARE+/^^−^ microbiome. This suggests that their healthy, established microbiome might resist colonization by an unhealthy microbiome exhibiting dysbiosis. One interesting observation is the identification of specific bacterial genera that seem to play a role in the inflammatory process driven by TNF: *Lactobacillus*, *Escherichia*, *Bacteroides* and *Parabacteroides*, *Actetanaerobacterium*, *Helicobacter*, *Clostridium* and *Lachnoclostridium*, *Eubacterium*, *Roseburia*, *Prevotella* and *Oscillospir*. Previous literature has shown that *Prevotella* might play a key role in RA, particularly *Prevotella copri* [10,33]. *Roseburia* has also been reported as an inflammatory signature in RA pathology [34]. *Prevotella copri* might be part of a causative pathobiont in RA [35,36]. *Bacteroides*, *Eubacteria*, *Acetanaerobacterium*, and *Lactobacillus* have also been shown to be RA footprints [37,38,39]. In addition, specific bacterial genera have been previously linked to inflammatory bowel diseases. Using 16s rRNA sequencing on pediatric patients with IBD, Hall et al. and Sila et al. observed increased prevalence of *Escherichia* and *Prevotella*, as well as decreased prevalence of *Bacteroides*, *Clostridium*, and *Roseburia* in the gut [40,41,42,43]. Wang et al. analyzed fecal and biopsy samples from IBD patients and found an increased prevalence of *Lactobacillus* [44]. There are now major indications of multiple bacteria acting together concomitantly in auto-immune disease etiology, dismissing the previously held idea that one bacteria or virus functions as the sole origin of auto-immune and inflammatory diseases [45]. However, the pathogenic mechanisms showing how gut microbiota dysbiosis in GF mice alters gut immune function and arthritis and IBD phenotypes remains to be elucidated. We collected a large amount of data, cells from the lamina propria of our mice gut and conducted flow cytometry and landscape modulating We found a strong landscape modulation of the innate lymphoid cell landscapes (ILC-1, ILC-2 and ILC-3 in line with the modifications observed in this taxonomic study, with some gender differences. Those data will be further analyzed and presented in a future immunologic publication.

For many auto-immune diseases, including RA and IBD, the specific causality has yet to be identified. In this particular study, the link joint-gut axis is interesting. Is the fact that both organs (joints and gut) are showing disease features, linked to the dysbiosis we observe? Could it be a direct effect, or an effect mediated by microbiota metabolites or other mediators? Some groups have discussed the link between the two pathologies [8,46] Brakenhoff et al. concluded that activated intestinal lymphocytes in IBD patients adhere to inflamed synovial vessels using multiple adhesion molecules and their counter receptors, of which VAP-1 supports the binding of all leucocytes. These finding provide an explanation for the pathogenesis of joint inflammation in IBD patients. Kontny et al. show that IBD-related SpA may originate from the relocation of the immune response primary induced in the gut associated lymphoid tissue, to the joints [47]. Thus far, we have used TNF-antagonists and other immunosuppressants to mitigate inflammation in auto-immune diseases such as RA and IBD. Those treatments are commonly used to treat both diseases, highlining the fact that they are mechanistically related. However, these treatments have shown variable responses in patients [48]. The complex mechanisms of the gut microbiome may account for this variability. The key player genera identified through our research could potentially act as autoimmune disease microbial biomarkers, leading to the development of diagnostic assays that will facilitate more personalized, and hopefully more effective, patient treatment plans. Additionally, fecal transplants are now being considered to treat select chronic and inflammatory diseases and IND submissions to the FDA are increasing [49]. In this context, it becomes important to understand and identify potential risks linked to fecal transplants in humans [50,51]. Our study shows that a healthy microbiome can dominate and normalize an unhealthy fecal transplant, limiting potential risks. Our findings may help with the regulation of FMT proposed therapies in humans, by the development of a potential treatment decisional tree. One major question remains: is gut microbiota dysbiosis a cause or a consequence of gut diseases such as IBD, associated with RA? Gut microbiome and IBD and RA literature review revealed that while there has been previous research on gut microbes associated with IBD and RA, there has been no conclusive evidence showing whether gut dysbiosis is the cause, or consequence, or even both, of autoimmune diseases. In a review published by Horta-Baas G et al., a whole section discusses microbiome and rheumatoid arthritis, with multiple studies showing a link between the gut microbiome dysbiosis and the pathogenesis of RA, and while causation is unclear, dysbiosis is a promoter for RA progression and contributor to arthritis maintenance [52]. Maeda et al. have shown that dysbiosis contributes to arthritis development via activation of autoreactive T Cells in the Intestine [53]. This study looked at whether arthritis-prone SKG mice would develop arthritis if inoculated with fecal samples from RA patients and found that the microbiome dysbiosis is a contributor to development of arthritis in genetically prone mice. The review by Ni J et al. asking the fundamental question of the causation or correlation, concluded that, while dysbiosis is linked to IBD in humans, it is unclear if dysbiosis is a cause or a consequence of IBD [54]. Finally, Wu et al. found that the microbiome dysbiosis was linked with RA, and dysbiosis promoted and/or exacerbated RA pathogenesis and inflammation [55].

We acknowledge that our study has some limitations: This is an observational study and the correlation between identified bacteria and TNF mechanisms involved in inflammation transmission need to be investigated and confirmed in other systems, along with the mediators involved. FMTs are designed to improve microbial dysbiosis and gut microbiome diversity in patients as a therapeutic approach, not to transmit a disease. We, however, wanted to approach not the treatment side, but the transmission side, exploring the role of microbiome in auto-immune diseases onset. Moreover, the use of heterozygous and not homozygous TNF^ΔARE−/−^ mice limited our results: the phenotype might have been stronger and more consistent over time with the use of homozygous donors. The features observed in degenerative cartilage disease might also account for the age of the colony: our mice survived until 6 to 9 months of age, when some arthritis degradation can naturally be observed. We also worked with relatively small colony numbers, because we encountered numerous challenges and deaths, since the mice were very fragile and difficult to maintain in an isolated environment.

## 5. Conclusions

Overall, our results confirm the involvement of the gut microbiome in the joint–gut axis and the potential role played by TNF in the inflammatory process and in disease etiology, driven by several specific microbes. Our results further open the door to a new field of research: investigating the interplay between the gut microbiome, the inflammatory process and disease etiology mediated via inflammatory mechanisms. Our future studies will involve the elucidation of mechanistic correlates between the microbiome key players identified in inflammation and autoimmune disease.

## Data Availability

All metadata have been deposited to NCBI microbiome database registry depository, in the Sequences Read Archives (SRA): https://www.ncbi.nlm.nih.gov/sra/ (accessed on 15 December 2021). Flow cytometry, Luminex raw data are available on demand.

## Supplementary Materials

The following are available online at https://www.mdpi.com/article/10.3390/microorganisms10010073/s1, Figure S1: title Study design.
