# Supplementary material for: Analyzing the Role of Gut Microbiota on the Onset of Autoimmune Diseases Using TNFΔARE Murine Model
Source: Microorganisms. 2021 Dec 30;10(1):73. doi: 10.3390/microorganisms10010073 (PMC8779571; doi:10.3390/microorganisms10010073)

Study design (including various controls)

- Mice are co-housed; Panel of controls (sentinels, C donors, IgG1)

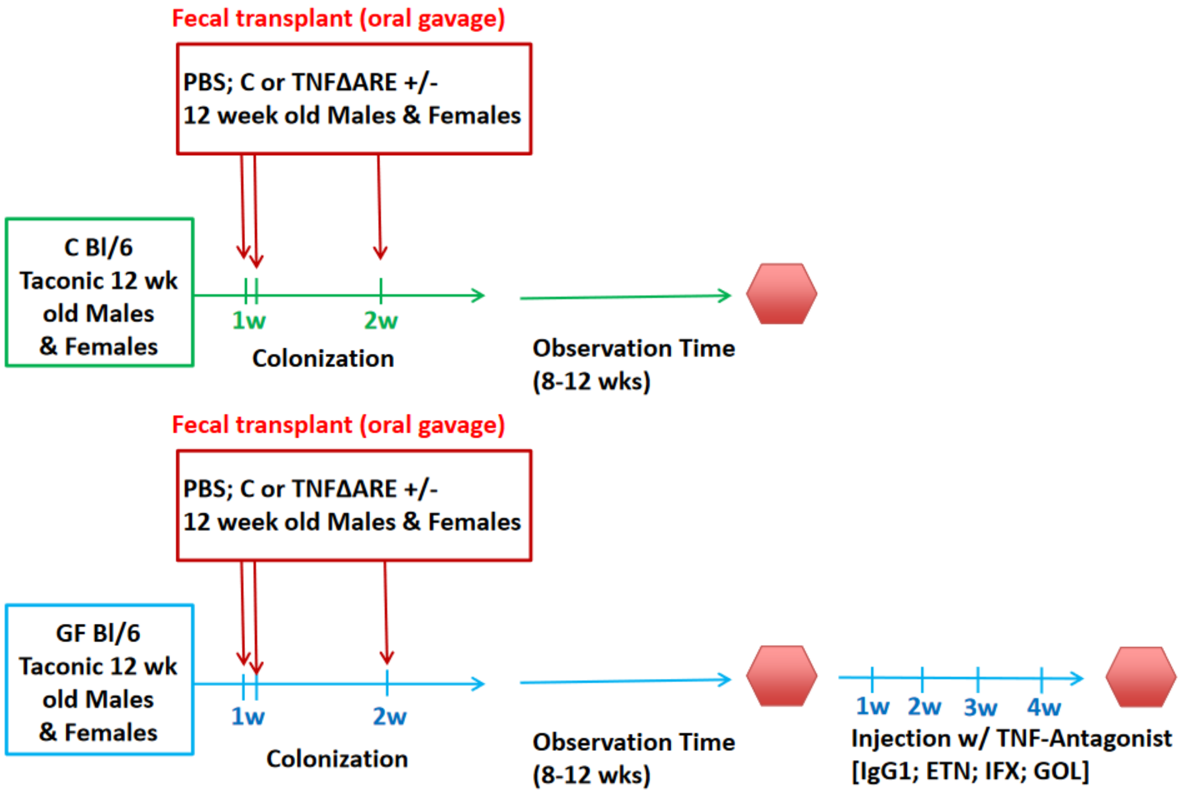

Supplement: Supplementary file 1 [file microorganisms-10-00073-s001.zip › microorganisms-1460022-supplementary.pdf]
